# Approval rates for corneal donation and the origin of donor tissue for transplantation at a university-based tertiary referral center with corneal subspecialization hosting a LIONS Eye Bank

**DOI:** 10.1186/s12886-022-02248-7

**Published:** 2022-01-10

**Authors:** Agata Anna Wykrota, Isabel Weinstein, Loïc Hamon, Loay Daas, Elias Flockerzi, Shady Suffo, Berthold Seitz

**Affiliations:** Department of Ophthalmology, Saarland University Medical Center (UKS), Kirrberger Straße 100, Building 22, 66421 Homburg/Saar, Germany

**Keywords:** Cornea, Eye bank, Organ donation, Organ donor card, Corneal transplantation, Keratoplasty

## Abstract

**Background:**

With the increasing demand for corneas, eye banks must optimize the tissue donation, collection, and selection process. This retrospective monocentric study analyzed the approval rates for corneal donation and the origin of and reasons for discarding donor corneas from 2010 to 2019.

**Methods:**

Data included the number of deceased, approval or rejection by the family for corneal donation and contraindications. Corneal grafts were included from all deceased persons who were full-body and multi-organ donors at the Saarland University Medical Center (UKS) and from external institutions. Additional analyzed parameters included endothelial cell count (ECC), blood sample serology for infections, and conjunctival swab testing .

**Results:**

A total of 1748 corneoscleral buttons were harvested from 10,265 deceased persons (17% with no contraindication) at the UKS between 2010 and 2019, with a consent rate of 23.3%. The number of explants increased from 136 in 2010 (15% of the deceased, total = 925) to 251 in 2019 (21%, total = 1214). Both the general and department-specific data showed similar percentages for corneal donation over the years, with intensive care and palliative units recently providing the most corneas. The increase in the number of corneas processed by the cornea bank over the years (368 in 2010 compared with 857 in 2019) was linked both to a better internal supply in 2010 (262, 71.2% of the total) compared with 2019 (519, 60.6%) and to an external supply by reinforcement of cooperation with external hospitals, including Luxembourg in 2010 (106, 28.8% of the total) compared with 2019 (338, 39.4%). A total of 195 of 377 corneas (52%) were discarded in 2009 compared with 260 out of 715 (36%) in 2019. The main reasons for discarding were low ECC (36% of discarded corneas in 2009; 11% in 2019), positive conjunctival swab (11% in 2009; 13% in 2019), and blood sample serology (6% in 2009 and in 2019).

**Conclusion:**

Despite an increasing number of donors, the demand for corneas is still rising. Improved cooperation with internal departments and with external clinics has led to an increasing number of explanted corneas. The main reason for discarding corneas was low ECC, followed by a positive conjunctival swab for fungal or bacterial contamination and serology. Increased donation rates and continued improvements in collection and selection processes are necessary to cover the high demand for corneas.

## Background

Organ transplantation is better known to the public than tissue transplantation despite an increase in tissue transplantation frequency [1, 2]. Transplantable tissues include cardiovascular tissues, musculoskeletal tissues, placenta (amniotic membrane), cells of the pancreatic islets and the human cornea [3].

Keratoplasty, introduced by Zirm in 1905 [4], is the oldest, most common, and most successful tissue transplant worldwide [5]. According to the World Health Organization, globally 1 billion people suffer vision impairment from various causes, including 4.2 million people presenting with corneal opacities [6].

In Germany, the removal of organs and tissues after death is permitted only if the deceased persons have given consent during their lifetime, or if their relatives have given consent on their behalf, considering the presumed will of the deceased [7, 8].

**Fig. 1:**
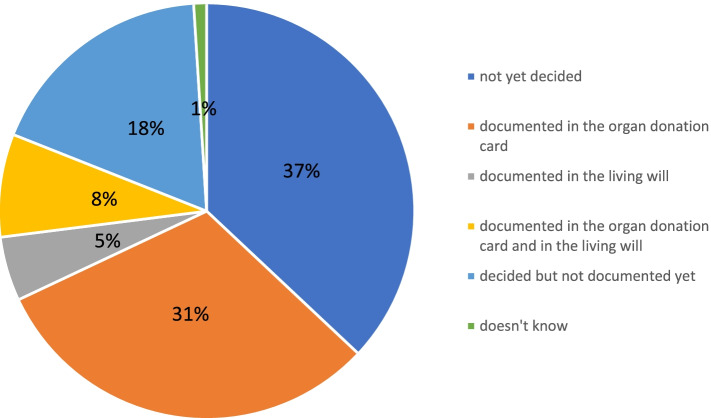
Results of the 2020 nationwide representative survey “Knowledge, attitude and behavior of the general population (aged between 14 and 75 years) for organ and tissue donation” run by the Federal Centre for Health Education on 4001 persons asked in Germany concerning the documentation of the decision to donate organs and tissues [9]

Since the first German organ donation cards were issued in 1971, the number of cardholders in the country has risen continuously, from 12% in 2001 to 39% in 2020 [7]. In a 2020 nationwide representative survey run by the Federal Centre for Health Education (Bundeszentrale für gesundheitliche Aufklärung) in Germany, 82% of more than 4000 participants showed a positive attitude towards organ and tissue donation [9]. However, only 44% of participants had documented their decision on an organ donor card or in a living will (Fig. 1). An internet-based survey among members of the German Ophthalmological Society (Deutsche Ophthalmologische Gesellschaft) concerning post-mortem corneal donation revealed that 79.4% of respondents would consent to corneal donation after death, but only 53.9% would be willing to obtain additional details on this subject [10]. In a different questionnaire among university hospital employees, 70.2% of the participants favored post-mortem corneal donation, whereas 57.4% said that they would appreciate more information [11]. Another multicenter survey of 5291 people questioned in various German cohorts showed that 65.2% expressed a willingness to donate [12].

**Fig. 2:**
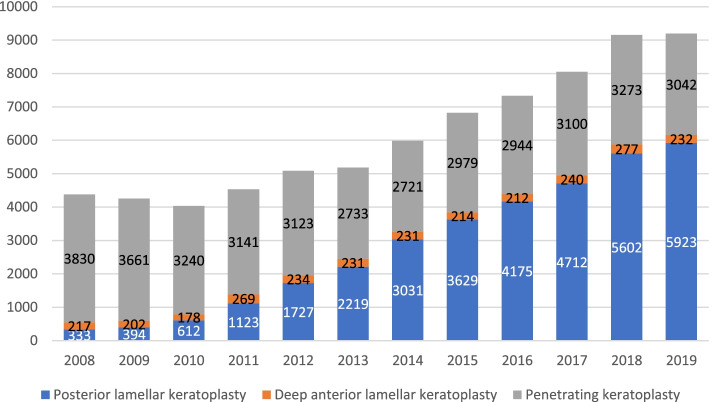
Number of lamellar and penetrating keratoplasties between 2008 and 2019 according to the German Keratoplasty Registry. **Note**: The term posterior lamellar keratoplasty includes both Descemet membrane endothelial keratoplasty and Descemet stripping automated endothelial keratoplasty

According to the German Keratoplasty Registry, the frequency of performed keratoplasties has increased every year in Germany since 2000, with more than 9000 keratoplasties in 2019 [13] (Fig. 2).

**Fig. 3:**
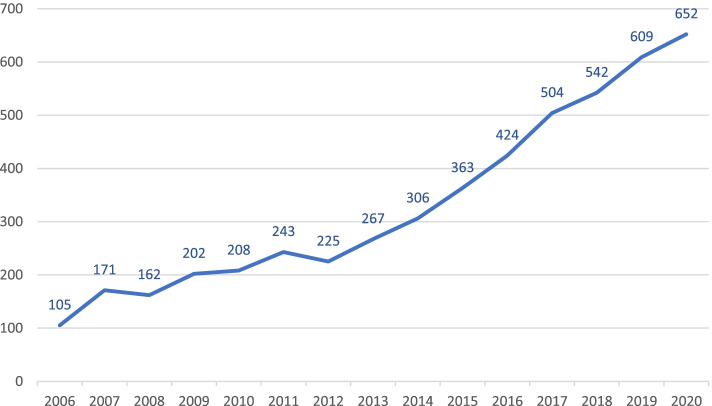
Total number of keratoplasties performed at the UKS Department of Ophthalmology between 2006 and 2020

According to the LIONS Eye Bank data, 609 corneas were transplanted in 2019 at the Department of Ophthalmology of the UKS in Homburg/Saar and 652 were transplanted in 2020 (Fig. 3).

In Germany, around 5000 patients are on the waiting list for keratoplasty. The demand for corneal tissue increases every year, making corneal donations crucial, but donations remain insufficient to meet need [14]. For this reason, eye banks must optimize the donation, collection, and selection processes for corneas. In this retrospective monocentric study, we analyzed approval rates for corneal donation and the origin of and reasons for discarding donor corneas from 2010 to 2019.

## Methods

### Settings and Design

In this retrospective, descriptive, monocentric study, we analyzed the approval rates for corneal donation, the origin of donor tissue for corneal transplantation and the cause for discarding corneas from 2010 to 2019 at the Klaus Faber Center, including the LIONS Eye Bank Saar-Lor-Lux, Trier/Westpfalz, within the Department of Ophthalmology, Saarland University Medical Center (UKS) in Homburg/Saar, Germany.

### LIONS Eye Bank of the UKS in Homburg/Saar (Germany)

The tasks of this institution are to obtain corneas from donors, ensure the quality of the tissue, and prepare the tissues for transplantation, including penetrating keratoplasty, posterior lamellar keratoplasty (including Descemet membrane endothelial keratoplasty [DMEK] and Descemet stripping automated endothelial keratoplasty), and deep anterior lamellar keratoplasty. Moreover, the Eye Bank is responsible for the acquisition and preparation of amniotic membranes, in cooperation with the UKS gynecology department, to be used as a graft, patch, or sandwich in cases of persistent epithelial defects and ocular surface inflammation [15].

### Sources of donor tissue for transplantation

The corneas transplanted in our facility are derived from deceased persons, including full-body donors in the Department of Anatomy [16, 17], multi-organ donors, and donors from external institutions in Germany or other countries (Luxembourg, the Netherlands, and Italy). To obtain corneas from the site of the UKS, the employees of the Eye Bank have a permanent internal access to online reports about the deceased, available since 2004 through the internal hospital information system, where deaths are reported in real time by each department According to the German Organ Transplantation Foundation (Deutsche Stiftung Organtransplantation), relatives are asked about the presumed will of the deceased if there is no organ donation card. In the absence of objections to the corneal explantation, a medical employee of the Eye Bank calls the department or the primary care physician to ask about the patient’s health history and to fill out the donor anamnesis protocol to determine possible contraindications (Table 1) inhibiting corneal removal. If there are no contraindications, the 15-mm corneoscleral button can be removed up to 72 h after cardiac arrest, according to the German regulations. However, explantation of donor corneas is usually performed within 24 h in our department because of the necessity of simultaneous blood sampling. The so-called “brain death problem”, the age of the deceased and a history of cataract surgery do not present obstacles regarding corneal donation. The donor corneas are replaced by a plastic shell and the eyelids are closed, leaving the facial expression unchanged. During the corneal removal, a blood sample and conjunctival swab are taken for examination. Because of the coronavirus disease 2019 pandemic, a nasopharyngeal swab for severe acute respiratory syndrome coronavirus 2 also has been taken since March 2020 [18]. These procedures are applied on all days, including non-working days such as weekends and holidays.

**Table 1:** List of the contraindications to corneal explantation

Unknown cause of death or significant disease of unknown etiology in the medical history	
Viral donor diseases: HIV (human immunodeficiency virus) infection, hepatitis B/C, HTLV I/II (human T-lymphotropic virus) or special risk factors for these infections	
Bacterial donor diseases: syphilis or other chronic persistent bacterial infections: brucellosis, typhus, rickettsiosis, leprosy, relapsing fever, melioidosis, tularemia	
Protozoonotic donor diseases: babesiosis, trypanosomiasis (e.g., Chagas disease), leishmaniasis	
Active systemic infections: bacterial, viral, fungal, parasitic or of unknown cause	
Fungal sepsis or sepsis with multi-resistant germs (a bacterial sepsis with the usual spectrum is not a contraindication)	
Disorders of the central nervous system (CNS) of unclear cause: Alzheimer’s disease, Parkinson’s disease, unclear rapidly progressing dementia, multiple sclerosis, amyotrophic lateral sclerosis, retroviral CNS disease	
Hematological neoplasias, leukemias, lymphomas	
Ophthalmic donor diseases with visible changes in the cornea, condition after corneal surgery, local infection, tumors of the eye	
Status after post-exposure vaccination against rabies within 12 months, status after live vaccines within 4 weeks	
Risk of disease transmission through prions: patients after dura mater-, cornea-, sclera-, hetero- or xenotransplantation, recipients of pituitary hormones, diagnosed Creutzfeldt-Jacob disease in the donor or in the family history	
Premortal use of substances that could have harmful effects on the recipient due to the transplant (e.g., poisons, heavy metals)	
Donors with premortal plasma thinning of more than 50% due to previous transfusions (plasma dilution up to 22.5 ml per kg of body weight is acceptable)	
Temporary exclusion: 2 years after healing of salmonellosis, Q-fever, tuberculosis, leptospirosis; 4 years after malaria cured; 4 weeks after measles, rubella, Varicella-zoster virus (VZV) or other serious viral diseases have healed	
Risk of ZIKV (Zika virus) infection	
Information about other unclear diseases; colonization or infection with Methicillin-resistant Staphylococcus aureus (MRSA)/ Extended spectrum beta-lactamases (ESBL)/ Vancomycin-resistant Staphylococcus aureus (VRSA)	
Indications of incomplete or unreliable medical history, doubts as to the accuracy of the information	
Additional information: natural death or confiscation by criminal police, diabetes mellitus, condition after chemotherapy and radiation therapy	
Coronavirus disease 2019 since March 2020	

The external facilities providing corneas included Luxembourg and collaborate clinics in Germany. Corneas were cultivated and prepared according to the protocol described below. To meet the demand for keratoplasties performed in our department, the Eye Bank also obtains corneas from cooperating Eye Banks from Aachen, Mainz, Venice, Rostock, the German Society for Tissue Transplantation (Deutsche Gesellschaft für Gewebetransplantation: DGFG), Bio Implant Services (BIS) in the Netherlands (human leukocyte antigen-matched corneas exclusively), and others. These tissues are ready for transplantation and are ordered on demand and availability.

### Preparation of donor corneas for transplantation

After explantation, corneas were placed in an organ culture medium, in two sequentially used formulations. Medium I was isotonic (307 mOsmol/kg) and consisted of 10% minimal essential medium, antibiotics (1% penicillin/streptomycin and 1% amphotericin B), 1% L-glutamine, 1.25% HEPES buffer, 3% NaHCO3, and 2% fetal calf serum. Medium II was formulated the same way but included the addition of a deswelling substance, e.g., dextran T500 6% (Arandal, Alchimia, Italy). The addition of dextran T500 6% alters the osmolarity of the organ culture medium from 307 mOsmol/kg to 353 mOsmol/kg, making the medium hyperosmotic .

At the time of explantation, the donor corneas were first placed into a transport medium, i.e., medium II. In the Eye Bank, corneas were transferred into organ culture medium I under strict sterile conditions, where they remained for a maximum of 28 days with medium exchange every 7 days. Before keratoplasty, corneas were transferred into organ culture medium II for deswelling for a maximum of 4 days. According to Hamon et al., corneas could be transplanted as soon as 12 to 24 h after transfer to medium II [19]. The corneas were stored at 34–37 °C. Endothelial cells were counted by specular microscopy, and slit lamp exams were performed twice during the cultivation period to detect scars or signs of previous surgeries. Information about cataract surgery, diabetes, chemotherapy, or causes of death such as sepsis in the donor history was obtained, although this information does not necessarily reduce the suitability of corneal donor tissue (Kramp et al. [20]). Sterile donor tomography has been routinely performed in donors corneas since 2018 to detect curvature abnormalities (e.g., keratoconus or status post-refractive surgery [21, 22]. After all of these procedures, tissues were approved to be transplanted.

### Data collection

This study included 10,265 deceased persons considered by the Klaus Faber Center, including LIONS Eye Bank Saar-Lor-Lux Trier/Westpfalz as potential cornea donors. For all deceased persons, approval or rejection rates for donation were analyzed. In case of approval, we analyzed the origin of the donor tissue, the presence or absence of contraindications and the results of the Eye Bank screening for endothelial cell count (ECC), conjunctival swabs, and serological analysis. We compared these results for each year between 2010 and 2019. Trends in corneal discarding as well as numbers of corneas delivered by external facilities were also analyzed over the years.

### Statistical analysis

The collected data included categorical variables, which are presented as absolute number and percentage.

## Results

### Development of approval rates

**Fig. 4:**
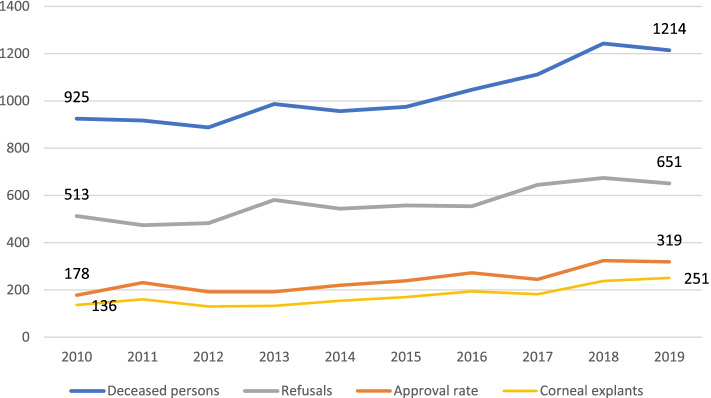
Number of deceased persons, refusals and approvals to the corneal donation and corneas removed (1 donor = 2 corneas) at the UKS in Homburg/Saar between 2010 and 2019

**Fig. 5:**
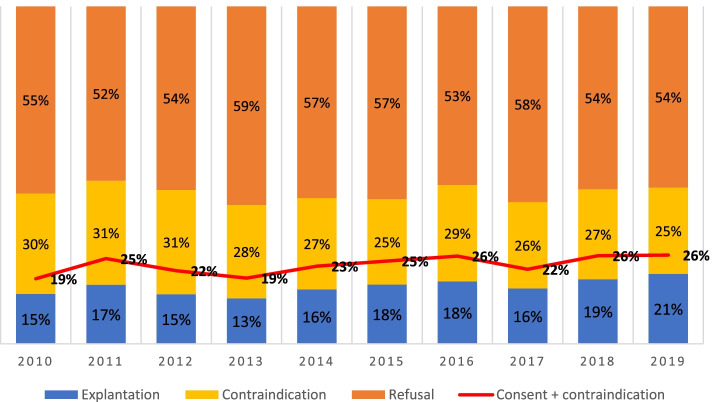
Percentage of cornea explantations, as well as contraindications and refusals to corneal donations in the years 2010–2019. The red line shows the percentage of consents of relatives to corneal explantation, including a small proportion of contraindications

Between 2010 and 2019, a total of 1748 corneoscleral buttons (17%) were removed from 10,265 deceased persons in the UKS. The number of deceased persons increased from 925 in 2010 to 1214 in 2019 (Fig. 4). The numbers of consent, refusals, and donations grew proportionately, peaking at 251 explants of 1214 deceased in 2019 (21%), compared with 136 of 925 in 2010 (15%) (Fig. 5).

**Fig. 6:**
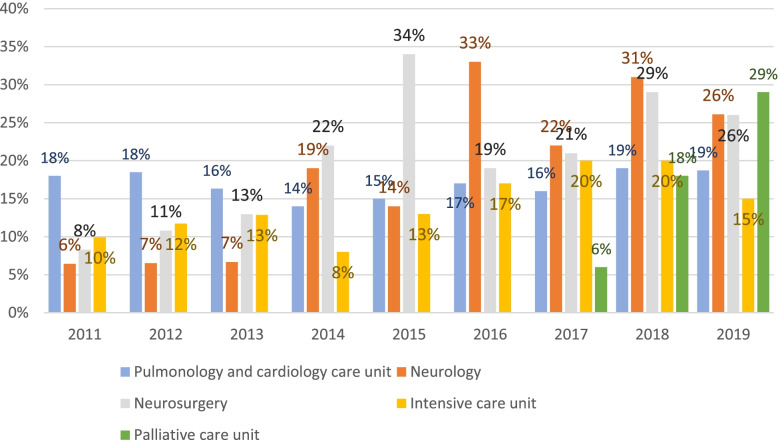
Data showing the units with most recorded corneal donations at the UKS – pulmonology and cardiology, intensive care, neurology, neurosurgery, and palliative wards. Note: The palliative unit exists since October 2016

Fig. 5 shows the number of consents and contraindications to the corneal explant. In some cases, contraindications for explantation existed despite approval, and these corneas were not explanted. The averaged values over this period were 23.3% for consent, 55.3% for refusal to donate, and 17% for removal itself. Both the general and department-specific data showed approximately the same percentages of corneal donation over the years. The pulmonology and cardiology, intensive care, neurology, and neurosurgery units, along with palliative ward (established in October 2016) had the most corneal donations. The trends among the departments are shown in Fig. 6.

### Development of corneal donations and tissue rejection

**Fig. 7:**
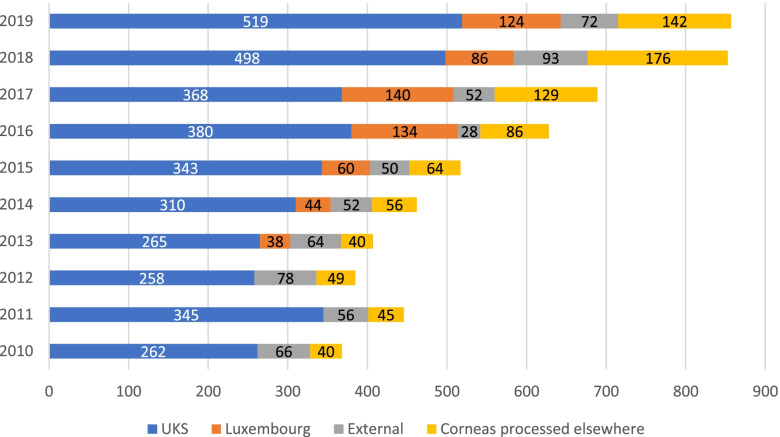
Number of donated corneas from UKS, Luxembourg and the external facilities to our Eye Bank, as well as corneas processed in other Eye Banks and bought by the UKS Eye Bank from 2010 to 2019. Note: The official cooperation with Luxembourg did not exist before 2013

The LIONS Eye Bank closely cooperates with other ophthalmological departments in Germany, Luxembourg (since 2013), Italy, Austria, and the Netherlands, as well as with the DGFG. Figure 7 presents the increasing number of donated corneas from the UKS, Luxembourg, and the external facilities, as well as corneas processed elsewhere since 2010 Fig. 8 shows the ratios of percentages of corneas obtained and processed at UKS together with corneas obtained from external facilities, including Luxembourg, in comparison with corneas processed in other eye banks and bought by the Eye Bank of the UKS Department of Ophthalmology during 2010–2019. The LIONS Eye Bank acquires processed ready-to-use corneal tissue from several facilities (Fig. 9).

**Fig. 8:**
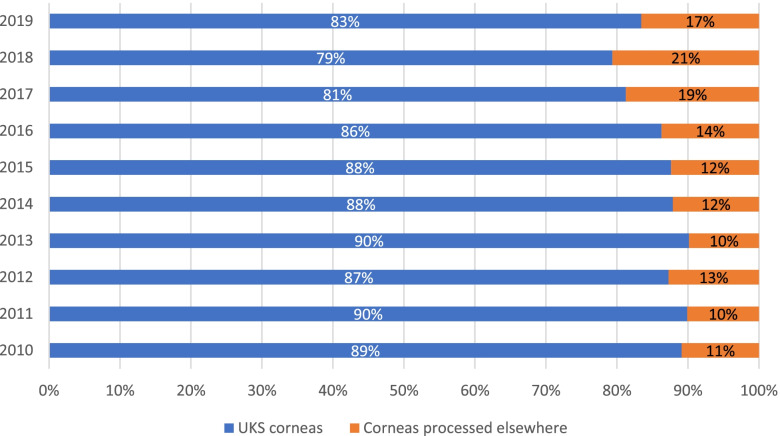
Percentage of the corneas obtained from UKS, and the corneas obtained from external facilities but processed in Homburg compared to the corneas processed in different Eye Banks and bought ready-to-use by the Eye Bank of the UKS in the years 2010–2019

**Fig. 9:**
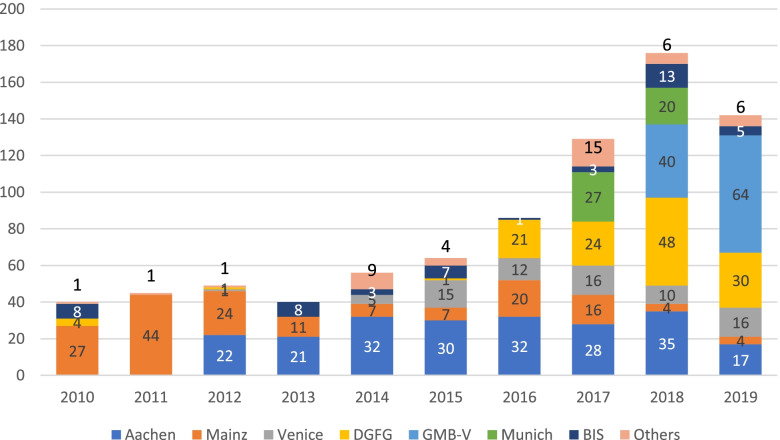
Number of corneas ready-to-use that have been bought from external Eye Banks in the years 2010–2019. BIS = Bio Implant Services; GBM-V = Gewebebank, Mecklenburg-Vorpommern, Rostock; DGFG = German Society for Tissue Transplantation (Deutsche Gesellschaft für Gewebetransplantation). Note: BIS is responsible for the human leukocyte antigen-matched corneas exclusively; number 6 – Others – represents delivery facilities not mentioned before

**Fig. 10:**
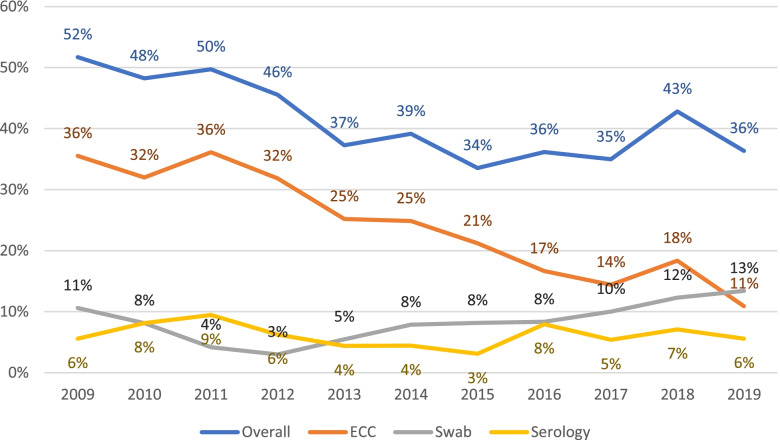
Percentage and reasons for discarding corneas since 2009 – overall number of tissue contamination, endothelial cell count (ECC), conjunctival swab and serology being the main reasons for corneal rejection

Since 2009, we have collected data on the reasons for corneal discarding, e.g., insufficient endothelial cell count, positive conjunctival swab, or positive serology with 195 of 377 corneas (52%) discarded in 2009, compared with 260 of 715 (36%**)** in 2019. The highest number of transplants suitable for surgery – 301 of 453 corneas (66%) – was documented in 2015 (Fig. 10).

## Discussion

The number of keratoplasties increases every year, and the number of patients on the waiting list for corneal transplantation continues to rise. According to the LIONS Eye Bank data, 609 corneas were transplanted in 2019 at the Department of Ophthalmology of the UKS in Homburg/Saar and 652 were transplanted in 2020 (Fig. 3). The number of deceased persons increased from 925 in 2010 to 1214 in 2019, while the proportion of consents has remained at around one quarter, resulting in 15–21% explantations over the last decade. In 2019, 359 people donated 715 corneas after their death, while there were 349 patients on the waiting list for a transplant in Homburg alone. Overall, around 5000 patients across Germany are still waiting for a donor cornea, and, the number of corneas donated at UKS obviously is not enough.

This situation explains why the trend is to acquire ready-to-use corneas bought from external facilities, as the percentage of corneas being prepared for transplantation by the Eye Bank has not kept pace. For various specific reasons, the department in Mainz was the major source of corneas at first, followed later by Aachen and DGFG, and most recently Rostock .

Several factors can influence the willingness to donate corneas and tissue suitability for transplantation. In the absence of a previously expressed wish for or against organ donation, the physician in charge of the treatment or a medical employee of the Eye Bank asks the deceased’s next of kin about the presumed will of the deceased. In a difficult situation of grief and despair, such a question can lead to additional stress, which may in turn influence the decision and result in the refusal of the corneal explant [11, 17]. In our study, an average of 23.3% of the family members of the deceased person agreed to the corneal donation, and 55.3% declined it. The emotionally difficult situation after the death of a close relative, together with limited knowledge about procedures for transplantation, brain death-related problems, and tissue suitability after the death of a potential donor, may lead to an unwillingness to donate [14].

A positive development is the much higher number of people with organ donor cards in Germany, at 39% in 2020 compared with 12% in 2004 [14]. Thus, an examination of decision trends in years is important. Of note, the willingness to donate may differ depending on socioeconomic group and country region, as analyzed by Uhlig et al. in three studies [10–12]. Their three separate analyses had something in common: most of the participants expressed an intention to donate corneas post-mortem as well as an interest in receiving additional information about donation and suggested the Internet as the most appropriate source of information on this subject.

As part of our selection process, we first obtained the consent of all potential donors and then clarified possible contraindications. This sequential procedure explains why not all corneas consented for harvest were harvested. Despite consent to corneal donation, we do not explant corneas after 24 h following death. German regulations, which are based on EU regulations, preclude taking the blood sample required for analysis after this time. Therefore, another factor in whether corneas are harvested can be a delayed report of a death to our Eye Bank, which would make the timely collection of the cornea impossible. For this reason, it is essential to continuously work on improving communication among different departments to avoid the loss of potential donors because of organizational problems.

All corneas were closely examined prior to their use in surgery. Examinations were done either at the LIONS Eye Bank or externally, depending on the tissue source. According to German regulations, the minimum ECC required is 2000 cells/mm^2^ for keratoplasty and 1000 cells/mm^2^ for urgent keratoplasty or anterior lamellar keratoplasty. In our ophthalmology department, we use only transplants with >2000 cells/mm^2^ for deep anterior lamellar keratoplasty because of the risk of intraoperative endothelial perforation with the need to convert the operation to penetrating keratoplasty [23], and we use >2200 cells/mm^2^ for DMEK. Additional requirements for DMEK are no donor diabetes and a donor age > 55 years. Kramp et al. examined 4140 corneas for factors possibly influencing the suitability and, in some cases, leading to discarding of donor corneas [20]. Based on their findings, information about cataract surgery, diabetes, chemotherapy or causes of death such as sepsis in the donor history should be obtained, although this history does not necessarily reduce the suitability of corneal donor tissue. These conditions as well as the results of the conjunctival swab and serology will define the final suitability of the tissue.

The introduction in November 2010 of a quality management system at the LIONS Eye Bank had a major impact on the rate and reasons for discarding corneas, thanks to improved standards, protocols, and training [24]. After evaluation of 4140 corneas between 2006 and 2016, Laun, Kramp et al. reported that the rate of discarded corneas because of poor endothelial quality, contamination of the medium, or positive conjunctival swab decreased significantly from 50.1 to 39.7%. Similarly, we found that 52% of corneas were discarded in 2009, compared with 36% in 2019. The main reason for discarding of corneas tended to be an insufficient ECC, followed by a positive conjunctival swab for fungal rather than for bacterial contamination and finally by the serology. However, the endothelial quality as the main cause of tissue rejection has shown a downward trend over the last decade, with 36% being discarded in 2009 and only 11% in 2019. The reasons for this continuous decrease are multifactorial. One factor could be additional quality controls at the Eye Bank [24] that tended to improve graft quality but could have resulted in discarding of the cornea prior to ECC measurement. Better ECC may be attributable to improved eye surgery techniques (minimally invasive procedures, endothelial protection using viscoelastics for cataract surgery) or to Eye Bank procedures such as cooling of donor bodies before cornea collection, shorter time between death and collection (typically <12 h versus 3 days in 2009), and an improved cultivation process (cleanroom since 2019). It is also plausible that compared with 2009, from 2015 we were able to better assess donor endothelium with a new inverted specular microscope (Primovert, Co. Zeiss) and an analysis tool assisting technicians in evaluating endothelial cells (REA XDL, Co. Robin), potentially leading to fewer discarded corneas. In addition, regular training of Eye Bank technicians who had been continuously replaced over the decade and the predominance of these technicians in doing the harvesting rather than less experienced residents could have resulted in less endothelial damage and thus less ECC-based discarding.

The conjunctival swab for fungi became a part of the diagnostics in 2014, as did a more detailed serology examination, including testing for hepatitis B/C, HIV, and syphilis. These results show how important critical selection and additional testing are to ensure the best transplant quality .

Li et al. analyzed the correlation between positive conjunctival swabs and microbial contamination of the culture medium, where donor corneas are held prior to transplantation [25]. They found a significantly higher contamination rate of the culture medium in cases of contaminated conjunctival swabs, which was subsequently a reason for discarding the tissue, as we found in the current work. The timing of post-mortem blood sample collection, the time between death and corneoscleral button excision, and storage time all seem to be important factors as well [20, 26]. Kramp et al. found that when blood sample collection took place more than 12 h after death, corneas were discarded more often because of positive donor serology [20]. According to Röck et al., a prolonged time from storage and death to corneoscleral button explantation is associated with an increased risk of tissue unsuitability for transplantation. Because we remove corneoscleral buttons up to 24 h after cardiac arrest, it could be beneficial to consider tissue removal in the first 12 h after death [26].

Compared to the findings of Schaub et al. who reported 235 (5.1%) corneas from 4593 patients, donated after death from 2011 and 2015 [27], we found that 1748 (17%) corneoscleral buttons were donated from 10,265 deceased persons in the UKS between 2010 and 2019. The increasing absolute number of corneas explanted in our department seems to be quite favorable and promising. This observed growth in corneal explants also may have resulted from the practice of performing corneal explants daily, including on non-working days. Doctors on duty during weekends or holidays checked the routine online report of deceased persons every morning through the internal hospital information system and, in the absence of objections to the corneal explantation, ensured that corneas were obtained right away.

## Conclusions

We found that 1748 corneoscleral buttons (17%) were taken from 10,265 deceased persons between 2010 and 2019 at UKS. The removed donor corneas were critically selected and tested for potential contamination before transplantation into people with eye injury or disease. The main reasons for discarding corneas were insufficient ECC, positive conjunctival swab and blood sample serology, with 52% of 377 corneas discarded in 2009 but only 36% of 715 in 2019. In addition, the number of excised donor corneas increased from 136 in 2010 (15%) to 251 in 2019 (21%). The demand for corneal tissue continues to grow, and making the choice to donate tissues post-mortem should be further popularised.

## Data Availability

The dataset supporting the conclusions of this article is available on request to the corresponding author.

## Abbreviations

BIS: Bio Implant Services
DGFG: German Society for Tissue Transplantation (Deutsche Gesellschaft für Gewebetransplantation)
DMEK: Descemet membrane endothelial keratoplasty
ECC: Endothelial cell count
GBM-V: Gewebebank, Mecklenburg-Vorpommern
UKS: Saarland University Medical Center
