# Correction to: Approval rates for corneal donation and the origin of donor tissue for transplantation at a university-based tertiary referral center with corneal subspecialization hosting a LIONS Eye Bank

**DOI:** 10.1186/s12886-022-02263-8

**Published:** 2022-01-24

**Authors:** Agata Anna Wykrota, Isabel Weinstein, Loïc Hamon, Loay Daas, Elias Flockerzi, Shady Sufo, Berthold Seitz

**Affiliations:** Department of Ophthalmology, Saarland University Medical Center (UKS), Kirrberger Straße 100, Building 22, 66421 Homburg/Saar, Germany

**Correction to: BMC Ophthalmol 22, 17 (2022)**

**https://doi.org/10.1186/s12886-022-02248-7**

Following the publication of the original article [1], we were notified of a mistake in the Abstract (marked in bold below):

The phrase: “The number of **keratoplasties** increased from 136 in 2010 (15% of the deceased, total = 925) to 251 in 2019 (21%, total = 1214).”

Should read: “The number of **explants** increased from 136 in 2010 (15% of the deceased, total = 925) to 251 in 2019 (21%, total = 1214).”

The original article has been corrected.
